# yMap: an automated method to map yeast variants to protein modifications and functional regions

**DOI:** 10.1093/bioinformatics/btw658

**Published:** 2016-11-21

**Authors:** Ahmed Arslan, Vera van Noort

**Affiliations:** KU Leuven, Centre for Microbial and Plants Genetics (CMPG), Leuven, Belgium

## Abstract

**Summary:**

Recent advances in sequence technology result in large datasets of sequence variants. For the human genome, several tools are available to predict the impact of these variants on gene and protein functions. However, for model organisms such as yeast such tools are lacking, specifically to predict the effect of protein sequence altering variants on the protein level. We present a python framework that enables users to map in a fully automated fashion large set of variants to protein functional regions and post-translationally modified residues. Furthermore, we provide the user with the possibility to retrieve predicted functional information on modified residues from other resources for example that are predicted to play a role in protein-protein interactions. The results are complemented by statistical tests to highlight the significance of underlying functions and pathways affected by mutations. We show the application of this package on a yeast dataset derived from a recent evolutionary experiment on adaptation to ethanol.

**Availability and Implementation:**

The package is available from https://github.com/CSB-KUL/yMap and is implemented in Python.

**Supplementary information:**

Supplementary data are available at *Bioinformatics* online.

## 1 Introduction

Non-synonymous Single Nucleotide Polymorphisms (nsSNPs) potentially disrupt normal protein functions. Several computational tools exist today that can predict the damaging effects of nsSNPs on protein structure and functions. For example, PolyPhen predicts the result of amino acid substitutions on the structure and function of human proteins (Adzhubei *et al.*, 2010). The impact of these tools is enormous on clinical interpretation of human nsSNPs but no such tool is available for the model organism yeast although SNPs are largely available and being identified in large-scale experiments. The most helpful tool to interpret nsSNPs in yeast is the SGD Variant Viewer (Cherry, 2015), but it does not predict impact on structure and function. Hence a tool to analyze the effect of nsSNPs on different functional regions of yeast proteins is much needed to accelerate research in yeast systems biology. We chose to develop such a tool and particularly focus on Post-Translational Modications (PTMs) and protein domains affected by nsSNPs. UniProt (The UniProt Consortium, 2014) contains over 200 different types of PTMs. PTMs can modulate biological interactions and broadly impact on protein functions, stability and localization hence phenotypes in response to stimuli. An nsSNP at a PTM residue under normal or experimental conditions could explain a change in phenotype and could thus be important for interpretation of effects of nsSNPs (Reimand *et al.*, 2015).

**Fig. 1.**
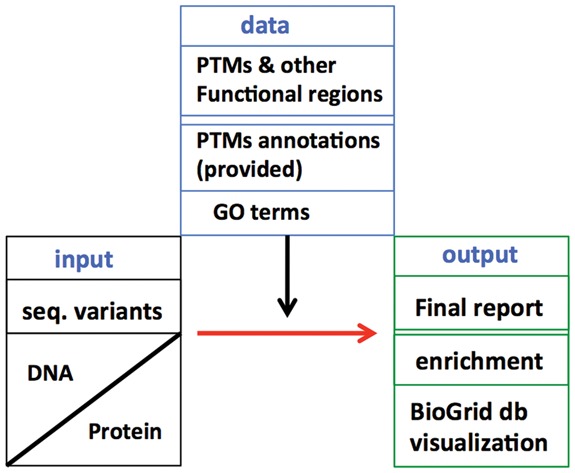
An overview of data acquisition and processing in yMap. The input sequence variants are provided by the user. Functional annotation is downoloaded by data() function. Mapping gives rise to individual reports and a final report

Yeast has been an excellent model to study systems biology. It helped considerably in understanding different molecular phenomena including protein-protein interaction and network biology. The exponential increase in yeast PTM data has left a gap between the identification and the functional annotation of PTM residues (Beltrao *et al.*, 2013). At the same time, due to the way they are stored it is not straightforward to map variations to the available annotated PTMs. Previously, a few studies have mapped residue variation or conservation to PTM residues (Minguez *et al.*, 2013, Reimand *et al.*, 2015). In order to facilitate researchers to map large datasets of yeast mutations onto PTM residues, we developed an automated method. A python module called yeast genotype to phenotype map (yMap) provides users with the possibility to map their variant data to PTM containing residues and protein functional regions (Fig. 1). We include mapping to functional and structural attributes of proteins, including protein domains, active sites, binding sites, protein-nucleotide binding motifs, structural regions and include a function to generate web links for pathways/networks visualization. The yMap package provides a stable and easy to use framework in pure python for functional analyses of yeast variants.

## 2 Features

Before you can initiate a variant analysis, you need to run a sub module of the yMap package to download and filter all the data needed to perform the mapping (detailed manual in Supplement). The major functions of the package can be categorized as following:

***Residue level annotation:*** The data() function downloads UniProt files for the yeast proteome, extracts annotations on residue level and stores these in a tab-separated file. It also downloads a gff file with the yeast genome sequence and annotation from SGD. Annotations of PTMs from PTMfunc (Beltrao *et al.*, 2012) and PTMcode v2.0 (Minguez *et al.*, 2015) are provided with the download. A user-provided file containing mutations (Supplementary Fig. S1) is converted to mutation types with the mutations-types-file() function, annotating synonymous, non-synonymous and nonsense mutations, protein residue number and reference and mutated amino acid. Finally, the ymap() function maps mutations to the residue level annotations. Files are generated for each separate category of functional annotations, as well as a summary file. This module returns different data folders, specific for each type of data, and contains files with mutations mapped onto the PTMs that are annotated for PPI, interface regions, present in proteins domains (Minguez *et al.*, 2015) interacting with other PTMs between different proteins or within the same protein (Beltrao *et al.*, 2012). Apart from PTMs, mutations are mapped to DNA binding motifs, active and binding sites, secondary structure regions.

***Pathway and network analysis:*** A separate file returns GO terms overrepresented among genes containing nsSNPs located in functional regions. The domain annotation is from UniProt. The p-values are calculated using Orange Bioinformatics against the background of the complete genome (Demšar *et al.*, 2013).

A file with BioGrid (Tyers *et al.*, 2006) IDs of mutated proteins is generated by ymap(). This module generates web links of these BioGrid IDs for visualization of pathways, networks and associated information available in the BioGrid database.

All the analyses are summarized in a *‘final-report’* with the proteins that have variants in different above mentioned protein functional regions, the type of mutation (synonymous or non- synonymous), feature, feature type and source of data. The report is complemented with enrichments file and a BioGrid ids containing file.

We have re-analyzed variants from evolutionary experiments on adaption to high levels of ethanol (Voordeckers *et al.*, 2015). In all experiments together, 1,207 non-synonymous and non-sense mutations were found in sequenced clones adapted to high ethanol. A subset of 232 mutations overlap with a protein functional region. One of these is a mutation of a phosphoserine (S391) in HAL5, a protein kinase with putative role in cell surface biosynthesis. A phosphoserine (S1358) in CHD1, an ATP-dependent chromatin remodeling factor, is found mutated to an asparagine. We identify 21 mutations in protein kinase domains such as VPS15, CDC7, CDC15 and RIM15. Together, this suggests adaption may be achieved through rewiring of the signaling network. Finally, mutations of PRT1, eukaryotic translation initiation factor 3 subunit B, are found within two structural regions. The K462E mutation in the same protein was found to increase ethanol tolerance in confirming experiments (Voordeckers *et al.*, 2015). Instead of selecting mutations based on frequency in the population, mutations can now be prioritized based on their expected impact on the protein level.

## 3 Conclusion

We present an automated method to analyze the impact of nsSNPs on protein level in yeast for the first time. The python package for yeast genotype to phenotype mapping yMap focuses on mutations in functional and structural protein regions. This package can be used to analyze mutations that affect important functional regions including PTMs, protein domains, active and binding sites, DNA-protein binding motifs, PPI, PTMs crosstalk and hotspots and the secondary structural regions. The method can facilitate the interpretation of large-scale variant data and the correlation of genotype with phenotype.

## Funding

This work has been supported by the KU Leuven Research Fund.

*Conflict of Interest:* none declared.

## Supplementary Material

Supplementary Data
