# Supplementary material for: yMap: an automated method to map yeast variants to protein modifications and functional regions
Source: Bioinformatics. 2016 Nov 21;33(4):571–3. doi: 10.1093/bioinformatics/btw658 (PMC5408805; doi:10.1093/bioinformatics/btw658)
Supplement: Supplementary Data [file btw658_supp.zip › SupplementaryInformationYMapv3.docx]

**Supplementary data**

**yMap: An automated method to map yeast variants to protein modifications and functional regions**

**Ahmed Arslan and Vera van Noort**

Centre of Microbial and Plant Genetics (CMPG), Department of Microbial and Molecular Systems (M^2^S)

KU Leuven, Kasteelpark Arenberg 22 –box 2460, 3001 Leuven, Belgium.

*Introduction:* The yMap package maps in a fully automated fashion large sets of variants to protein functional regions and post-translationally modified and suggests potential pathways altered through these mutations. The package takes the genomic variant data from high-throughput experiments; either these data consist of genomic coordinates with reference and alternative base or protein level mutations with mutational positions (both types of files are included in the example_mutation_file folder)

**Contents:**

**1 – Manual**

**2 – How to use yMap package on Yeast Ethanol experiment data from Voordeckers et al 2015**

**1 Manual *- Start working with yMap package:***

The package can easily be installed from the Python Package Index by using package manager *pip* with the following command.

**$ pip install ymap**

It takes three small steps to perform the analysis (step4 is optional), which takes mutational data and convert them to functional data. Before performing the mutation analyses, the functional annotation data need to be downloaded locally (step1) with the command ydata().

**Step1: $ ydata**

After the first step, the ymap folder should contain the following files; these files are needed for the subsequent mutation mapping (we also summarize their properties and contents in the README file):

uniprot_mod_raw.txt (contains raw data from UniProt)

gff.txt (contains chromosome sequences from yeast genome)

yeastID.txt (contains yeast proteins ids)

uniprot_bioGrid.txt (contains yeast proteins BioGrid ids)

PTMs.txt (contains yeast PTMs positions and types)

PTM_id_file.txt (contains different proteins ids map onto PTMs.txt)

domains.txt (contains yeast proteins domains and positions)

id_domain.txt (contains yeast proteins ids map to domains.txt)

nucleotide.txt (contains yeast proteins-nucleotide binding motifs)

id_nucleotide.txt (contains yeast proteins ids map to nucleotide.txt)

pdb.txt (contains yeast proteins sec. structure and positions)

bact.txt (contains yeast protein active and binding site positions)

frmt.txt (contains format gff.txt with genomic coord. and st. orient.)

d_id_map.txt (contains protein ids map to frmt.txt)

sc_within_proteins.txt (PTMcode 2.0 [2]- contains PTMs present within a protein)

sc_btw_proteins.txt (PTMcode 2.0 - contains PTMs present between proteins)

3DID_aceksites_interfaceRes_sc.txt (PTMfunc [3] - acetylation present at protein interface)

3DID_phosphosites_interfaceRes_sc.txt (PTMfunc – phosphorylation present at protein interface)

3DID_ubisites_interfaceRessc_sc.txt (PTMfunc – ubiquitination present at protein interface)

SC_acet_interactions.txt (PTMfunc – acetylation involved in PPi)

SC_psites_interactions_sc.txt (PTMfunc – phosphorylation involved in PPi)

SC_ubi_interactions_sc.txt (PTMfunc – ubiquitination involved in PPi)

schotspot_updated.txt (PTMfunc - contains PTMs present in hotspots)

More details about these files can be read in the README file in ymap package. Now all the data is available to perform the ymap analysis on the user provided mutation file. The user should copy the mutation file to the present directory or the folder where the user wants to save the results files (step2). Please make sure the requirement data and mutation file are located in the same directory.

**Step2: copy the “mutation file” to the present directory**

The following command(s) performs the mutational analyses data on the user provided mutation file (step3). If the input file contains protein level mutations, the following command should be used (see “example_mutation_file/mutation.txt” file):

**Step3: $ yproteins**

Or

if the input file contains gene level mutations with chromosomal positions, the following command should be executed (see” example_mutation_file /mutated_proteins.txt”):

**Step3: $ ygenes**

Note: The mutation file name should be the same filename as mentioned above for the respective data type. That is, “*mutated proteins.txt*” name for gene level mutation file and “*mutation.txt*” for proteins level mutation file.

After executing the above command, the user should have a folder “yMap-results” which contains a number of subfolders (see below) and a *final-report.txt*, this report sums up all the data results from all the analyses run in ymap (fig S1). The first column is the uniprot ID of the mutated protein, common name, mutation position, ref. amino acid, mutated amino acid, protein feature-type mutated, protein feature (region) and data source. The *biog.txt* file contains BioGrid ids of mutated proteins, the user can run**, $ yweb**; upon executing this command, the user will be asked to provide the path of directory (yMap-results) contains biog.txt file, /path/to/biog.txt*, which in turn tells the program where to look for the file. This command leads to an html based visualization of the network of proteins with mutated functional regions in BioGrid db ((fig S2-A)). A third file contains the GO enrichments of genes containing non-synonymous mutations in functional regions where the whole genome is used as background (fig S2-B)


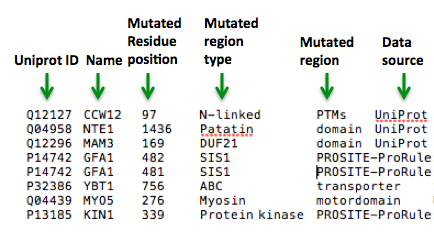


**Fig S1:** overview of the final-report file from ymap() method.

The output of step3 is a folder with the name of the protein feature it represents as following subfolders:

…./yMap/ PTMs

…./yMap /Domains

…./yMap / A-B-sites

…./yMap / PDB

…./yMap / Nucleotide_binding

…./yMap / PPI

…./yMap / Interface

…./yMap / PTMs_hotSpots

…./yMap / PTMs_between_Proteins

…./yMap / PTMs_within_Protein

Each of the subfolders contain three types of files, one, with mutated proteins (Fig. S3-A; in this case, the file contains the mutations overlap with the domains regions), second, *pvalue.txt* with enrichment results of each analysis (Fig. S3-B) and third, *biog.txt* with BioGrid ids of mutated proteins for networks and pathways visualizations via web (Fig. S3-C).


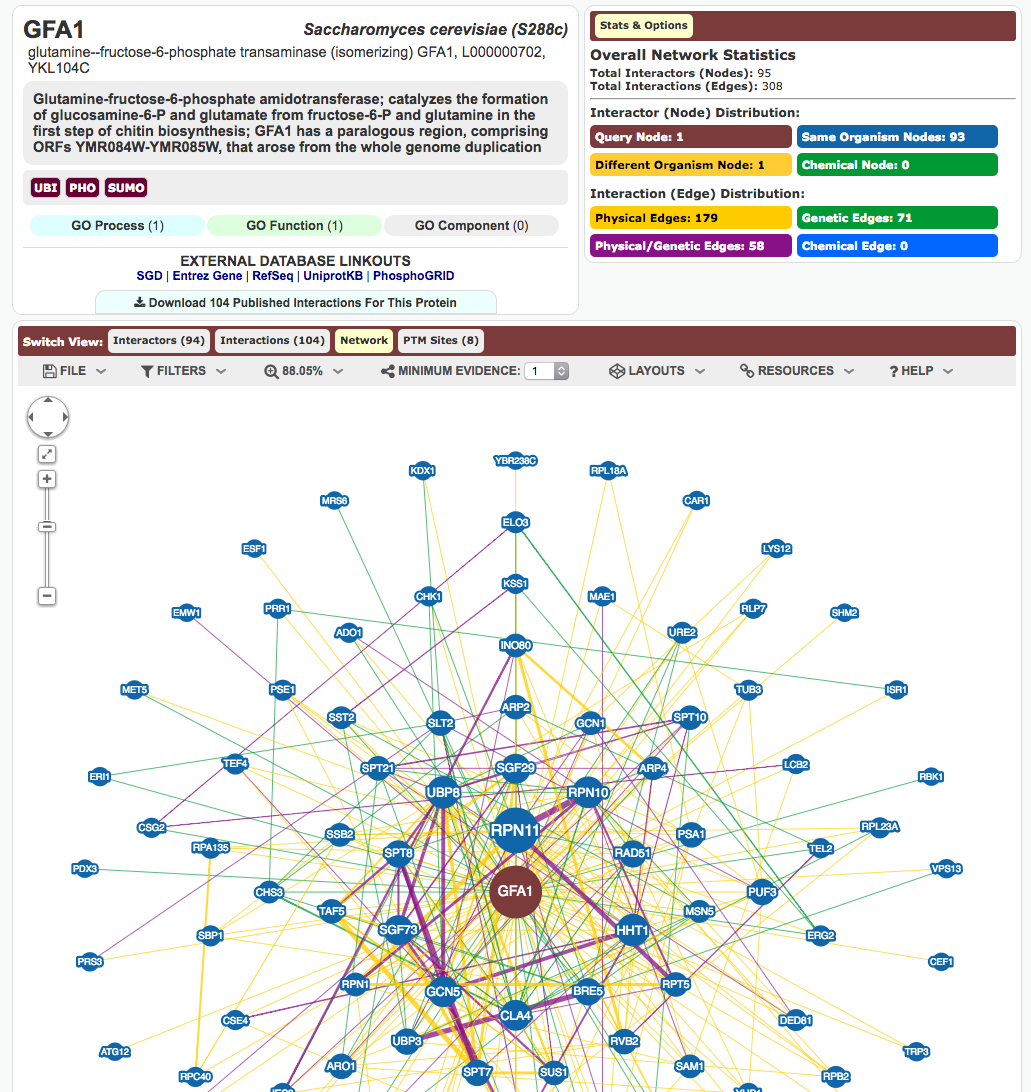


(B)

'amino sugar biosynthetic process', 0.015047021943573663, 'GFA1'

'nucleotide biosynthetic process', 0.046684350132626, 'GFA1'

'glutamine-fructose-6-phosphate transaminase (isomerizing) activity', 0.015047021943573663, 'GFA1'

**Fig S2:** The y*web* methods functionalities (A) the example page of BioGrid db for mutated protein in analysis

and following sub-folders (B) a *pvalue* file returns the enrichments of all the non-synonymous mutations in ymap analysis

(A)


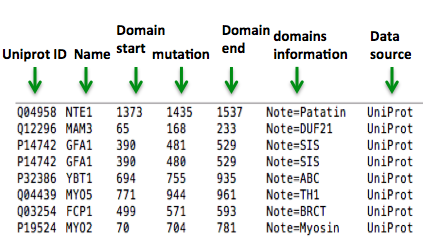


(B)

'organophosphate catabolic process', 0.02939393939393938, 'NTE1'

'glycerolipid catabolic process', 0.02871821664925113, 'NTE1'

'cellular biogenic amine metabolic process', 0.03496865203761755, 'NTE1'

(C)


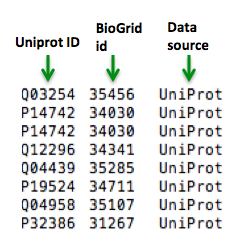


**Fig. S3:** The outcome of a method in ymap, in this case, (A) the results “domains” folder comprises of mutations falls in proteins domains, (B) the enrichment scores with pathways, p-value and proteins names (C) the BioGrid ids for networks visualization as in Fig S2-A.

(To run ymap from source code, please see the README file at package’s homepage.)

**2 – How to use yMap package on Yeast Ethanol experimental evolution data from Voordeckers et al 2015**

To exemplify the functionalities of the ymap package and also to facilitate the users of ymap, we analyzed the example data from Voordeckers et al 2015, below is described how to use the data.

The data was obtained from the supplementary information of the article (<http://journals.plos.org/plosgenetics/article?id=10.1371/journal.pgen.1005635#sec029>), the data is in text file format, we did not need to change its format. These data can also be found in the Supplementary-analyses zip Archive Folder *DataVoordeckersetal*. This folder contains 12 files with mutation data. For each of the six fermentors mutations were identified in individual clones and in the whole population (eg F1_clone.txt contains mutations identified in individual clones from Fermentor 1).

The input data files were stored in a folder, and requirement data to perform ymap were downloaded with the command ydata(), since ymap is executable from any directory we change to the directory where we want to save our results. We made sure the requirement data is in the present directory where we want to store the output (results).

**Step1: $ ydata**

The following command was run on each of the mutation files one by one to analyze all the data from Voordeckers et al 2015. Since the input data is of the type genomic coordinates, before executing step3 of program, we changed the file names to “mutated_proteins.txt”. In the end, all the data was analyzed and saved in the individual folders and also supplied as attachments to supplementary information with this article.

**Step 2: > cp F1_clone.txt mutated_proteins.txt**

**Step3: $ ygenes**

The final report (final_report.txt) after analysis of F1_clone.txt looks like this:

P38970 HAL5 391 Phosphoserine PTMs UniProt

P28274 URA7 321 Glutamine amidotransferase type-1 PROSITE-ProRule:PRU00605domain UniProt

P32571 DOA4 294 Rhodanese PROSITE-ProRule:PRU00173domain UniProt

P39109 YCF1 559 ABC transmembrane type-1 1 PROSITE-ProRule:PRU00441domain UniProt

P43565 RIM15 1738 Response regulatory PROSITE-ProRule:PRU00169domain UniProt

P0CH08 RPL40A 31 Ubiquitin-like PROSITE-ProRule:PRU00214domain UniProt

P48510 DSK2 339 UBA PROSITE-ProRule:PRU00212domain UniProt

P53746 FRE4 432 FAD-binding FR-type PROSITE-ProRule:PRU00716domain UniProt

P06103 PRT1 373 PDB:4U1F Helix UniProt

P48510 DSK2 339 PDB:2BWF Helix UniProt

Q06142 KAP95 728 PDB:3EA5 Helix UniProt

P38131 KTR4 314 PDB:5A07 Helix UniProt

P53051 IMA1 482 PDB:3AJ7 Helix UniProt

P10964 RPA190 500 PDB:4C2M Helix UniProt

The first line indicates that a Phosphoserine (pos 391) of HAL5 is found mutated. The Phosphoserine annotation is derived from UniProt annotation. In the supplementary zip archive, this file can be found in >Clone >F1 and is called *final_report.txt*. The individual reports for different functional regions are also found in this folder. The file *mutation.txt* contains annotated mutations, that is gene name, protein name, reference and mutated amino acid, type of mutation, chromosome and position.

*Note: A user has to provide the path to the directory containing the biog.txt as input after –w option. In python 2.x, the path is “path/to/biog.txt” but in python 3.x it’s without inverted commas, path/to/biog.txt
